# Production of ^15^N-Labelled Liquid Organic Fertilisers Based on Manure and Crop Residue for Use in Fertigation Studies

**DOI:** 10.1371/journal.pone.0150851

**Published:** 2016-03-16

**Authors:** Belén Martínez-Alcántara, Mary-Rus Martínez-Cuenca, Carlos Fernández, Francisco Legaz, Ana Quiñones

**Affiliations:** 1 Department of Citriculture and Vegetable Production, Instituto Valenciano de Investigaciones Agrarias, Moncada (Valencia), Spain; 2 Institute of Animal Science and Technology, Valencian Polytechnic University, Valencia, Spain; Yeungnam University, REPUBLIC OF KOREA

## Abstract

Large quantities of crop residue and animal manure from agricultural and livestock activities are annually produced worldwide. With proper management, these residues are potentially valuable sources of plant nutrients, mainly N. Recycling such subproducts in sustainably-based agricultural systems can minimise the use of mineral fertilisers, and hence reduce the potential risk of surface and groundwater pollution. Therefore, the purpose of this study was to obtain (small scale) two liquid labelled-organic fertilisers, an animal- and a vegetal-based organic (AO and VO, respectively) fertiliser, to be used as organic N sources in subsequent fertigation studies. Forage maize (*Zea mays* L.) grown under ^15^N-labelled fertiliser supply was used as raw material for VO fertiliser production, and also as ^15^N-labelled sheep feed to obtain ^15^N-labelled manure. The labelled faeces fraction was used as raw material for the AO fertiliser. The VO fertiliser was obtained after an acidic and an enzyme-driven hydrolysis. The AO fertiliser was obtained after acidic hydrolysis. The VO liquid fertiliser presented an N concentration of 330 mg·L^-1^, 85% of total N was organic, while ammonium and nitrate N accounted for 55% and 45% of the mineral nitrogen fraction, respectively. This fertiliser also exhibited high K, Ca and S concentrations and notable values for the remaining macro- and micronutrients. The AO liquid fertiliser had a similar total N concentration (496 mg·L^-1^, 82% of total N in an organic form) to that of VO, but its mineral N fraction significantly differed, which came in a predominantly (95%) ammonia form. It also had a high content of N, P, K and other macronutrients, and sufficient Fe, Zn, Mn, Cu and B levels, which suggests its suitability as a potential fertiliser. The percentage of ^15^N enrichment in both VO and AO liquid fertilisers exceeded 2% ^15^N atom excess, which enabled their use in subsequent assays run to assess nitrogen uptake efficiency.

## Introduction

Large quantities of crop residue and animal manure generated by agriculture and livestock are annually produced worldwide. Today’s annual USA production of agricultural residue is around 500 10^6^ dry Mg [1]. In China, approximately 800·10^6^ Mg of agricultural residue are produced annually [2], while a similar value (approximately 700·10^6^ Mg) is obtained in the EU [3]. Regarding animal manure, the produced quantity depends on animal species, diet, age, the environment and productivity, among other factors, with values ranging from 30 to 25,500 kg·year^-1^ for broilers to lactating cows, respectively [4]. The total population in the EU amounts to 136 10^6^ livestock units, of which 48% is bovine and 14% is poultry [5]. With proper management, this residue is a potentially valuable source of plant nutrients, mainly N. Its potential reuse as organic fertilisers could represent a sustainable approach to recycling nutrients and reintegrating organic matter into soil [6]. Recycling such nutrients in ecological and sustainably-based agricultural systems could not only minimise the use of mineral fertilisers, but could also reduce the potential risk of surface and groundwater pollution with nitrates. The objectives and targets of European legislation have been key drivers to improve residue management, to stimulate innovation in recycling and to limit landfilling use (EU, 2010). However in order to successfully recycle agricultural and livestock organic residue as a nutrient source, release of N and its uptake efficiency must be clearly identified. This is essential information because farmers are unable to consider organic residue a nutrient source when calculating fertiliser doses [7]. It would also help shed light onto the misleading perception that manure is an unreliable nutrient source [8]. Nevertheless, it is extremely complicated to predict the availability to crops of residue derived-N because it must be mineralised by soil microbes before being made available for its uptake [9]. This also varies vastly depending on the estimation method [8], and is dependent on the characteristics and “quality” of organic residue [10]. Some approaches based on the difference in N uptake in the presence and absence of added residue have been carried out, but poorly precise measurements (residue derived-N is a small fraction of total crop N uptake) and the impossibility of tracing N in soil pools greatly limit the information acquired from these indirect determinations [11].

In line with this, accurate residue-derived N tracing can only be achieved by using ^15^N isotope-dilution techniques. The ^15^N labelling technique has been previously used as a tool to assess the fate of manure N in long-term N cycling studies with daily cattle [8,12], goat or pig [13,14,15], poultry [16] and sheep [17,18,19] manure. Some research works have used this technique for the ^15^N-labelling of plant residue [20,21,22]. However, these assays obtained organic residue in a solid form, which must be solubilised and mineralised before becoming plant-available [17]. Moreover, any nutrients present have a limited short-term effect on crop nutrition [23,24], but significantly contribute in crop N requirements during the year after applying manure. Given the implementation of more efficient irrigation systems (drip irrigation, subsurface irrigation or sprinkler systems), this solid organic residue cannot be used as a fertiliser without undergoing prior hydrolysis processes to increase its solubility to enable its use in fertigation.

Very few works on the production of liquid fertilisers using crop residue or animal manure have been published. Capulín-Grande et al. [25] assessed the production of liquid cattle manure extracts to study their subsequent contribution to *Lolium perenne* plant nutrition. Liquid fertilisers obtained from seaweed have been used in India and China for decades [26]. These liquid fertilisers have shown high levels of all trace elements, plant hormones and vitamins, but negligible amounts of N and P [27]. Similarly, a manure tea made by fermenting cow dung in water has been reported as a moderate NPK fertiliser and a major iron source [28,29] to evaluate the nutritional component of the liquid fertilisers that derived from three common plants. All liquid manures had a higher nutrient content than common solid organic fertilisers.

Therefore, the purpose of this study was to obtain two liquid ^15^N-labelled organic fertilisers (small scale), an animal and a vegetal-based organic fertiliser, to be used as a source of N and other essential elements to assess their efficiency as potential sources of N and other micro- and micronutrients in citrus fertigation in subsequent experiments.

## Material and Methods

### Ethics statement

15N-labelled sheep manure production was carried out in the Institute of Animal Science and Technology (Valencia Polytechnic University, Spain). The experimental procedure was approved by the Animal Use and Care Committee of the Valencia Polytechnic University, and followed the codes of practice for animals used in experimental works proposed by the EU [30]. The remaining experiments were conducted in the Department of Citriculture and Vegetal Production of the Valencian Institute of Agrarian Research (IVIA, Moncada, Spain). Dr. Ana Quiñones was response for the experimental analyses in this manuscript and can be contacted in the future. The authors declare that this manuscript neither contains any ethic issue nor involves endangered or protected species.

### ^15^N-labelled forage maize production

Forage maize was used as raw material to obtain 15N-labelled animal and vegetal-based liquid organic fertilisers as it is one of the main cereals grown in the EU (EUROSTAT, 2015) and will, therefore, lead to larger amounts of crop residue.

In August 2009, forage maize (*Zea mays* L.) was grown in three plots, 10 m x 2 m each, on sandy-clay soil (67.5% sand, 10.6% silt, 21.9% clay; pH 7.9 and 0.58% organic matter content) at the IVIA centre (39°55´N, 0°39´W) in Moncada (Valencia, Spain). Plots were irrigated by microsprinklers and a nutrient solution that contained the necessary macro- and micronutrients. Six foliar extra-N applications were carried out fortnightly, three with a 0.5% (w/w) solution of (NH_2_)_2_CO (urea) and three with a 1.0% (w/w) solution of (NH_4_)_2_SO_4_ (ammonium sulphate, AS). Solutions were ^15^N-labelled (10 atom % ^15^N in excess). As a result, plots were fertilised with 495 g urea and 1,220 g AS, which accounted for 483.9 g N (80.6 kg N ha^-1^) and 48.4 g ^15^N in excess. Before applying the fertiliser, a plastic film was placed at a depth of 50 cm to avoid ^15^N leaching loss.

In October, before plants started to flower, the aerial part of maize was cut and the root system was manually extracted. The whole root system was manually removed by pressurised water and by extracting small roots by hand. Plant tissues were separated into stems, both with and without leaves and roots. The water content of the leafy stems was reduced to 20% at room temperature to stabilise the plant matter to be used for hay for sheep feed. The other fractions were dried in an oven at 65°C and representative subsamples were stored at 4°C until the mineral concentration and nitrogen (^15^N/^14^N) isotopic compositions were further analysed.

### The sheep-feed ^15^N-labelling procedure

In order to increase the ^15^N enrichment of sheep feed, 43.8 kg of stems and leaves were sprayed with a solution that contained 200 g of ^15^N-labelled urea (10.2% atom ^15^N excess). Extra labelled leafy stems were mixed and divided into 18 feed rations to ensure uniform ^15^N enrichment in the daily feed supply [8].

### ^15^N-labelled sheep manure production

The forage method, by which ruminant livestock manure is labelled by feeding ^15^N-enriched forage [12], although costly and laborious [17], it is the only available method for labelling urinary and faecal N components in the present-day, but ensures sufficient enrichment for subsequent organic fertiliser production.

Four dry sheep were used to produce labelled manure. Loss of ^15^N in milk was thus avoided because between 20% and 30% of the N (protein) fed to a ruminant is converted into milk [8]. Sheep were housed in metabolic cages 7 days prior to the labelled feeding phase to give them time to acclimatise, and were fed with unlabelled straw to adapt them to the diet. Labelled maize (stem with leaf fractions) was administered for 18 days (from 22 February to 11 March 2010), offered *ad libitum* and renewed every 48 h. Uneaten maize was removed from the feeder before providing each new supply.

Urine and faeces were collected daily from cages separately since the ^15^N enrichment of urine N was significantly lower than that of simultaneous faecal N [17]. Urine was collected from tubes that drained into containers fixed in ice to minimise gaseous N loss, and was then stored at 4°C. Faeces were hand-scraped from the perforated platform of the metal cages, weighed, oven-dried at 65°C and dry-weighed for dry matter determinations. Faeces and urine from each collection were subsampled and stored at 4°C for further analyses.

### Obtaining liquid ^15^N-labelled organic fertiliser from plant residue

Labelled maize fractions (500 g each) were mixed and ground in a vegetable shredder (Viking GE345), and further blended in a water-refrigerated mill (IKA M20, Staufen, Germany). The mixture was hydrolysed (cooking step) with 0.5% H_2_SO_4_ at high pressure (1.5 MPa) and temperature (125°C). The sulphuric acid that came into contact with organic tissue destroyed it and facilitated its solubilisation by releasing the contained elements [25]. Finally it was washed to prevent the acidic solution from continuing the subsequent process, and was filtered to separate the solid and liquid (Liquid Fraction 1).

**Fig 1:**
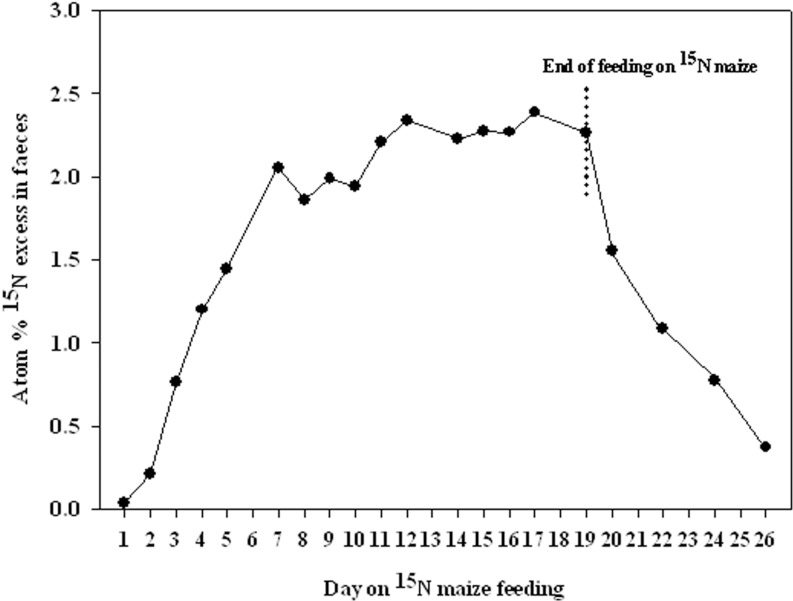
Schematics of the production liquid ^15^N-labelled organic fertiliser process from the ^15^N maize residue.

A 3-litre volume of water at 55°C was added to the solid fraction and pH was adjusted to 8.5 using NaOH. The resulting mix was subjected to action carbohydrate-degrading enzymes a multi-activer β-glucabase preparation produced by a selected strain of *Humicola insolens*, in which cellulase, xylanase, pentosanase and arabanase activities predominated. The hydrolysed fraction was filtered by producing Liquid Fraction 2 and a solid substrate. Protein hydrolysis was then performed in an alkaline medium in the solid fraction, which was resuspended in 4 L of water and adjusted to pH 3.0 with hydrochloric acid. Phytase (0.2 g and 6,000 units·g^-1^) and an acid fungal protease (0.4 g), both produced from *Aspergillus niger*, were added and the solution was stirred at 50°C for 20 h. The entire slurry was heated to 95°C for 20 min, cooled down and adjusted to pH 5 with NaOH. Filtering led to a solid residue and Liquid Fraction 3. All the obtained extracts (Liquid Fractions 1, 2 and 3) were then mixed to achieve an extraction of around 75% of the original plant matter (Fig 1).

### Obtaining liquid ^15^N-labelled organic fertiliser from animal residue

Labelled faeces were dried in an oven (65°C) and, after grinding as reported for the plant residue, were subjected to acid hydrolysis to enhance the N availability found in faeces for plants [24]. Faeces were digested in sulphuric acid (1.0%) at high pressure (1.5 MPa) and temperature (150°C), according to the method described by Capulín-Grande et al. [25]. These authors stated that addition of sulphuric acid to cattle manure led to higher P, Ca, Mg and S concentrations than natural liquid cattle manure extracts, or when stabilised by a warming-up process. After hydrolysis finished, extracts were decanted and the obtained fluid was filtered.

### Analytical determinations

Subsamples of the ^15^N-labelled maize and faeces were oven-dried (65°C) and ground in a water-refrigerated mill (IKA M20, Staufen, Germany) to pass through a 0.3 mm-diameter sieve. They were stored at 4°C until further analyses. In the vegetable samples, faeces and organic fertilisers, determinations of the total N concentration and ^15^N abundance were made in an Elemental Analyzer (NC 2500, Thermo Finnigan, Bremen, Germany) coupled to an Isotope Ratio Mass Spectrometer (Delta Plus, Thermo Finnigan, Bremen, Germany). The N in the urea/ammonium and nitrite/nitrate forms present in urine was determined by the Kjeldahl method. Macro- and micronutrients were measured by simultaneous ICP emission spectrometry (iCAP-AES 6000, Thermo Scientific. Cambridge, UK). The results were expressed as a percentage (macronutrient) or parts per million (micronutrients) of dry weight (DW). All the determinations were made in duplicate. After each set of 10 (in the N analysis) or 15 (for the other elements) samples, a standard was run to ensure accuracy.

With the liquid organic fertilisers, an analysis of mineral nitrogen (nitrate and ammonium) was measured by a flow injection analysis (Aquatec 5400, Foss Tecator) following the methodology described by Raigón et al. [31]. The analysis of the mineral nitrogen fractions (N-NH_4_^+^-NO_3_^-^) was measured by the flow injection analysis (FIAstar 5000, Foss Tecator, Höganäs, Sweden). In order to determine isotopic composition, liquid fertilisers were steam-distilled (2200 Kjeltec, Auto Distillation Unit, Foss Tecator, Höganäs, Sweden). ^15^NH_4_^+^-N and ^15^NO_3_^—^N were recovered in boric acid 0.32 N and reduced to dryness in an oven (P Selecta Barcelona, Spain) at 65°C. Aliquots were acidified with H_2_SO_4_ before the analysis done in the above-mentioned mass spectrometer [32]. Organic nitrogen content was calculated as the difference between total and mineral N contents by the Kjeldahl method, as described by Bremner [32].

### Calculations

The ^15^N content in forage maize, organic residue and fertilisers obtained was calculated as follows:

N15sample(mg)=N(%,w/w)⋅DW(g)⋅atom%N15excess⋅10−1

where *atom %* ^*15*^*N excess* was calculated by subtracting the natural abundance of ^15^N from the ^15^N excess of each sample. The natural abundance of ^15^N was considered the abundance of atmospheric N_2_, 0.3663 atom %, according to the International Atomic Energy Agency [33].

Total ^15^N recovery represents the proportion of applied or fed ^15^N (source), which is taken up by maize, is incorporated into faeces and urea, or is present in the fertilisers obtained.

Nrecovery(%)=N15maize,faeces,urine(mg)⋅100N15excess(mg)

## Results and Discussion

### ^15^N-labelled forage maize production

The biomass of the maize plants extracted at the end of the labelling period and the composition of the different fractions (leafy stems, leafless stems and root system) are shown in Table 1. The total maize biomass was 65.1 kg and leafy stems represented the main fraction (84% of the total DW). From the whole produced biomass, 25,743 mg of ^15^N in excess were recovered from the labelled fertilisers, which represents 53.2% of the ^15^N supplied (Table 1). This indicates that foliar N mineral fertilisation is a very efficient process to not only fertilise crops, but to also produce enough labelled plant matter for its subsequent use as raw material for organic fertilisers. In a field assay, Powell et al. [8] also found that 36–44% and 26–65% of ^15^N-ammonium sulphate (10 atom % ^15^N) was recovered in alfalfa and corn, respectively, in a 4-year experiment. Bosshard et al. [24] recovered 54% of the originally applied ^15^N in ryegrass shoots when N was applied as K^15^NO_3_ (15.9 atom % ^15^N).

**Table 1:** DW, N and ^15^N content of the total maize biomass produced at the end of the labelling period, and the fractions used as sheep feed and as raw material for the vegetal-based organic fertiliser*.

		DW	N	N	^15^N	^15^N
		(kg)	(% DW)	(g)	(% excess)	(g)
Labelled maize	Leafy stems	54.6	1.52±0.14	829.9±72.3	2.81±0.05	23.32±0.12
	Leafless stems	5.4	0.92±0.17	49.7±9.9	2.68±0.04	1.33±0.02
	Root system	5.1	0.65±0.01	33.2±5.6	3.29±0.07	1.11±0.04
	Whole plant	65.1	1.40±0.13	912.8±73.8	2.82±0.07	25.74±0.09
Sheep feed	Leafy stems ingested	38.9	1.86±0.11	723.5±21.2	3.75±0.14	27.13±0.26
	Surplus feed	4.9	1.86±0.11	91.1±18.6	3.75±0.14	3.42±0.15
Maize for fertilizer^†^		26.2	1.29±0.11	341.1±63.2	3.09±0.15	10.54±0.04

* Each value is a mean of three samples ± standard error.

^†^ Maize fractions used as raw material for vegetal-based organic fertiliser production: leafy stems not ingested (leafy stems-leafy stems ingested)+ leafless stems +root system.

The leafy stem fraction, mostly allocated from total plant ^15^N (91%), resulted in an isotopic enrichment of the N pool in this compartment of 2.82 atom % ^15^N excess. The root system showed remarkable higher ^15^N enrichment (3.29 atom % ^15^N excess), and its contribution to increase the average ^15^N enrichment of the whole plant (2.82 atom % excess) was negligible given its low proportion of total biomass (8%). In a similar assay conducted to produce ^15^N-labelled sheep manure, Sørensen et al. (1994) obtained ^15^N-labelled grass hay with 4.52 atom % ^15^N excess after 54 days of ^15^N-KNO_3_ supply (5 atom % ^15^N). Bosshard et al. (2011) reported a similar pattern in Italian ryegrass (14.6 atom % ^15^N in excess) labelled with K^15^NO_3_ (15.9 atom % ^15^N). It is noteworthy that comparatively higher enrichments were obtained in these assays because ryegrass was cultivated in sand and no isotopic dilution effect by soil native N took place.

The macro- and micronutrient concentrations of the leafy stems fraction (Table 2) indicated that the source of vegetable organic fertiliser presented high potassium levels and sufficient quantities in the other nutrient elements.

**Table 2:** Macro- and micronutrient concentration of the maize used as raw material for obtaining the vegetal-based organic fertiliser at the end of the growing cycle*.

Macronutrients (%)	P	K	Mg	Ca	Na	S
	0.04±0.04	0.70±0.08	0.05±0.01	0.22±0.04	0.02±0.00	0.05±0.01
Micronutrients (ppm)	Fe	Zn	Mn	Cu	B	
	186.0±23.3	30.8±2.5	10.9±2.4	2.4±0.7	13.5±3.7	

* Each value is a mean of three samples ± standard error.

### Labelled sheep feed

Part of the leafy stem labelled fraction (43.8 kg) was used as sheep forage. Supplementary ^15^N-urea labelled spray increased the ^15^N enrichment of sheep feed on 3.75 atom % excess. This supplemental mineral N ensured greater isotopic enrichment of the subsequent sheep faeces, and also represented 13.7% of the total N in sheep feed. This was clearly below the threshold (20%) of the total dietary mineral N, which can cause toxicity.

### ^15^N-labelled sheep manure production

Extra labelled leafy stems were supplied as sheep forage; 38.9 kg were ingested by animals and 4.9 kg were collected from troughs as residual (Table 1). During the ^15^N-labelling period, any uneaten hay was removed from the feeder before placing each new supply.

**Fig 2:**
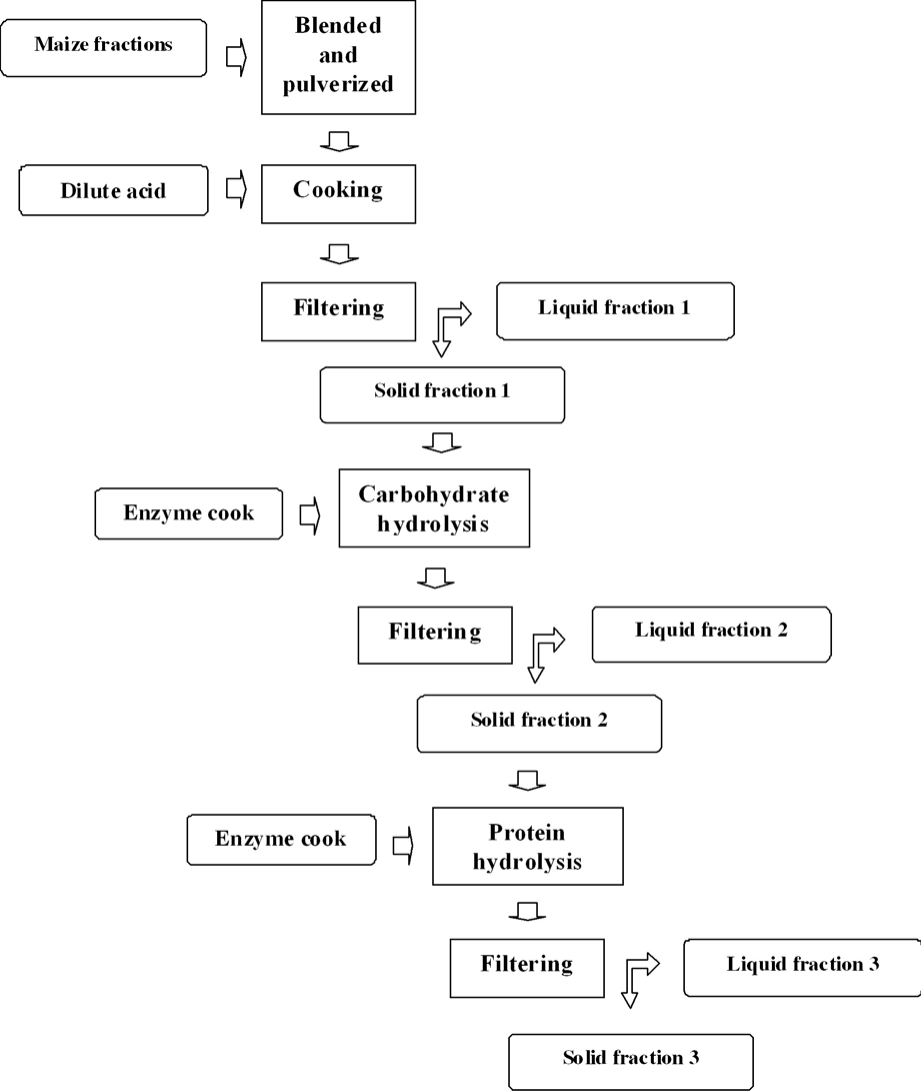
Time course evolution of the ^15^N enrichment of faeces from the sheep fed by ^15^N-labelled forage maize hay.

The ingest of labelled maize resulted in the increased ^15^N enrichment of faecal-N over time (Fig 2). On the initial days of feeding with the ^15^N-labelled feed, the ^15^N in faeces scarcely exceeded natural abundance (0.3663 atom % excess). This was because it was diluted by unlabelled indigestible feed N, which remains in the digestive tract, and by endogenous N, which consists in microbial products and microorganisms from the rumen, intestine and hind gut and N that originate from the digestive tract [8,34,35]. Subsequently, the ^15^N in faeces continues to be diluted by partly labelled endogenous N [17].

From day 3 of feeding on ^15^N, the ^15^N concentration considerably increased to reach a plateau value of 2.28 atom % excess between days 11 and 19, which coincided with the end of the feeding period on ^15^N hay. Similar results have been obtained by Sørensen et al. [17] from sheep fed ^15^N-labelled hay (4.52 atom % ^15^N excess) and Chantigny et al. [14] by feeding pigs an ^15^N-enriched diet (2.81 atom % ^15^N). These authors obtained ^15^N enrichment of 3.70 and 2.32 atom % after 9 and 12 days of labelled feeding, respectively. According to these authors, homogeneous labelling of sheep manure was achieved after a 7-day feeding period with ^15^N-labelled hay, which is in line with the results presented herein. At the end of isotope-enriched feeding, 58% of the ^15^N initially present in the labelled leafy stem fraction was recovered in sheep faeces. Brosshard et al. [24] recovered about 77% of the ^15^N in feed (ryegrass) in sheep manure.

From day 17 onwards, the labelled diet was replaced with normal unlabelled hay. Once the labelled feed was replaced with unlabelled fed, the ^15^N percentage in faeces dramatically lowered and, 22 days after starting the labelled diet, ^15^N enrichment barely exceeded natural abundance.

The manure obtained between days 7 and 19 of labelled feeding (Fig 2) accounted for 12.4 kg (DW), with 1.79% and 2.18% of N and ^15^N excess, respectively (Table 3). These labelled faeces were subsequently used to obtain an animal-based organic fertiliser, which presented ^15^N enrichment above 1.9%. These values (quantity and enrichment) enabled the use of the organic fertiliser obtained as a labelled product for subsequent trials based on the isotope dilution technique. The faeces that corresponded to previous and latter days (days1 to 6 and days 21 to 26) were discarded for their low ^15^N enrichment (less than 1% on average).

**Table 3:** Dry weight (DW), volume (V), N and ^15^N content of the different faeces fractions from sheep fed with an ^15^N-labelled maize diet*.

Source fertilizer materials		DW / V	N	N	^15^N	^15^N
		(kg / L)	(% DW / mg L^-1^)	(g)	(% excess)	(g)
Faeces discarded		9.3	1.86±0.10	172.8±9.6	0.91±0.12	1.57±0.03
Urine	N-(urea+NH_4_^+^)	27.9	4450±980	124.4±20.6	1.16±0.57	1.45±.0.04
	N-NO_3_^-^	27.9	0.35±0.14	0.01±0.00	0.85±0.23	0.00±0.00
Faeces for organic fertilizer		12.4	1.79±0.19	222.3±19.3	2.18±0.03	4.85±0.06

* Each value is a mean of three samples ± standard error.

At the end of the ^15^N-enriched feed period, the ^15^N recovered in solid manure accounted for 18% of the ^15^N present in sheep forage. This percentage increased to 29% when the discarded faeces and urine were counted (Table 3). The rest of the labelled-N was probably in the N assimilated by sheep. Higher values were found in the urine and faeces of the cows (51–64%) fed with enriched forage [8].

At the end of the labelled feeding period, the average isotopic enrichment of sheep urine was lower than that obtained in faeces (Table 3), and was always below 2% (data not shown) during the collecting period. Urine was, therefore, ruled out for obtaining the animal organic fertiliser. Powell et al. [8] also reported greater enrichment in faeces than in urine in cows fed ^15^N hay. Two days after starting ^15^N feeding, the ^15^N enrichment in faeces exceeded that in the urine ^15^N excess in other studies conducted with sheep [24].

As in forage maize, macro- and micronutrient concentrations were also determined in the solid manure (Table 4). The faeces used for the animal-based organic fertiliser generally obtained higher concentration values for most sampled nutrients (N, P, Mg, Ca, S, Fe, Zn, Mn, Cu and B) compared to forage maize, with similar levels for the potassium element as in the labelled forage maize. Therefore, sheep faeces proved also to be a major potential source of plant nutrients.

**Table 4:** Macro- and micronutrient concentration of faeces from the sheep fed an ^15^N-labelled maize diet used as raw material for obtaining animal-based organic fertiliser*.

Macronutrients (%)	P	K	Mg	Ca	Na	S
	0.37±0.05	0.92±0.11	0.25±0.03	0.82±0.08	0.05±0.01	0.21±0.03
Micronutrients (ppm)	Fe	Zn	Mn	Cu	B	
	309.5±22.1	185.9±15.8	65.5±5.6	9.6±0.2	38.3±3.3	

* Each value is a mean of three samples ± standard error.

### Obtaining liquid ^15^N-labelled organic fertiliser from plant residue

Uneaten hay, this being labelled maize leafy stems that were not extralabelled with sprayed urea, leafless stems and roots were used to obtain the ^15^N-labelled organic fertiliser from plant residue. The maize used as raw material presented 1.29% N and 3.09 atom % ^15^N in excess (Table 1).

The resulting vegetal-based liquid fertiliser presented high nitrogen concentrations (Table 5), mostly in an organic form (85%), while ammonium and nitrate-N respectively accounted for 55% and 45% of the mineral N fraction. The isotope enrichment of the resulting vegetal-based liquid fertiliser was sufficient for further uptake efficiency studies. The obtained liquid organic fertiliser also had high K, Ca and S concentrations, and showed notable values for the other macro- and micronutrients. The obtained plant-based liquid fertiliser retained 43% of the N originally present in the maize fractions used as raw material (Tables 1 and 5).

**Table 5:** Comparative volumes, total N and the N concentrations among fractions, ^15^N excess and N recoveries of liquid vegetal- and animal-based organic fertilisers (mg·L^-1^)*.

Fertilizer	V (L)	N total (mg·L^-1^)	N-organic (mg·L^-1^)	N-NH^+^_4_ (mg·L^-1^)	N-NO^-^_3_ (mg·L^-1^)	^15^N excess (atom %)	^15^N content (g)	Recovery (%)
Vegetal-based^†^	520	330.8±31.6	282.6±6.0	26.5±0.6^1^	21.6±0.0	2.62±0.07	4.51±0.10	42.7±3.6
Animal-based^‡^	135	495.7±47.1	403.3±0.0	88.2±0.4	4.2±0.4	2.17±0.01	1.45±0.05	29.9±2.1

* Each value is a mean of three samples ± standard error.

^†^ Ammonium and nitrate nitrogen accounted for 55 and 45% of released total inorganic nitrogen, respectively.

^‡^ Ammonium and nitrate nitrogen accounted for 95 and 5% of released total inorganic nitrogen, respectively.

### Obtaining liquid ^15^N-labelled organic fertiliser from animal residue

As previously indicated, solid manure with enrichment above 1.9% atom ^15^N excess was used as raw material to obtain the animal-based liquid fertiliser.

The obtained liquid animal-based fertiliser was characterised; N_total_, ammonia, nitric and organic N concentration and atom % ^15^N in excess, and the main macro- and microelements, were determined (Table 6). The animal-based organic fertiliser showed a total N concentration of 496 mg·L^-1^, mostly (82%) as organic forms. The mineral-N fraction, which accounted for only 18% of the total N present in the fertiliser, mainly came in an ammonia form (95% of mineral N). These results support those obtained by Jackson-Smith et al. [36], who obtained much smaller amounts of ammonium and higher concentrations of organically bound N in semisolid dairy manure than in slurry.

**Table 6:** Comparative values of macro- and micro concentrations in the liquid vegetal- and animal-based organic fertilisers (mg·L^-1^)*.

Fertilizer	P	K	Mg	Ca	S	Fe	Zn	Mn	Cu	B
Vegetal-based	47±1	923±14	60±2	386±9	548±7	8.3±0.3	2.5±0.5	1.2±0.0	0.13±0.00	0.16±0.00
Animal-based	365±26	921±65	257±19	545±28	1274±68	22.6±2.1	18.0±1.4	5.7±0.5	0.04±0.01	0.52±0.04

* Each value is a mean of three samples ± standard error.

The percentage of ^15^N enrichment in the animal-based liquid fertiliser was 2.17% lower than that in the vegetal-based one (2.62%). In this cooking, the process to obtain liquid fertiliser was less efficient, and about 30% of the N present in labelled faeces was recovered in the final fertiliser, where the by-products of the decanting and filtering stages were the main points of ^15^N loss. Carballo et al. [37] also managed to obtain a biofertiliser from a compost extract and their nitrogen recovery was about 55% of the ^15^N applied as labelled faeces. In order to increase the efficiency of this process on an industrial scale, the use of appropriate enzymes or microbe inoculums for hydrolysis or fermentation processes should be considered. A concentration step would also be of interest in order to cut the expenses associated with transporting the final liquid fertiliser.

The animal-based organic fertiliser had a similar potassium concentration to that of the vegetal-based organic fertiliser. The animal manure extract showed higher concentrations of the other nutrients than those obtained in the liquid plant fertiliser, which was expected in view of the raw materials analysis results (Tables 3 and 4).

## Conclusions

Processes to obtain two liquid fertilisers from crop residue and animal manure were assessed. Both fertilisers showed ^15^N enrichment that enabled their use in a subsequent experiment to assess N uptake efficiency, and to hence evaluate them as potential N sources in citrus fertigation. Their liquid presentation would also allow them to be used in more efficient irrigation systems, such as drip irrigation. The presence of other macro- (P, K) and micronutrients (Fe, Zn, Mn, Cu and B) suggests their suitability as potential multinutrient fertilisers.
